# Handgrip Strength Is Associated with Standing Long-Jump but Not 20-m Shuttle-Run Performance in Saudi Schoolgirls

**DOI:** 10.3390/children13101380

**Published:** 2026-10-12

**Authors:** Hessa A. Alhabib, Maha H. Alhussain, Rizwan Qaisar, Osama Aljuhani, Khalid Saeed, Abdullah G. Alshehri, Shaea A. Alkahtani

**Affiliations:** 1 Department of Food Science and Human Nutrition, College of Agriculture and Food, Qassim University, Buraydah 51452, Saudi Arabia; h.alhabeb@qu.edu.sa; 2 Department of Food Sciences and Nutrition, College of Food and Agricultural Sciences, King Saud University, Riyadh 11451, Saudi Arabia; mhussien@ksu.edu.sa; 3 Department of Basic Medical Sciences, College of Medicine, University of Sharjah, Sharjah 27272, United Arab Emirates; rqaisar@sharjah.ac.ae; 4 Space Medicine Research Group, Research Institute of Medical and Health Sciences, University of Sharjah, Sharjah 27272, United Arab Emirates; 5 Cardiovascular Research Group, Research Institute of Medical and Health Sciences, University of Sharjah, Sharjah 27272, United Arab Emirates; 6 Department of Physical Education, College of Sport Sciences and Physical Activity, King Saud University, Riyadh 11451, Saudi Arabia; oaljuhani@ksu.edu.sa; 7 The In Silico Drug Discovery Group, University of Helsinki, 00170 Helsinki, Finland; khalid.saeed@helsinki.fi; 8 Biomechanics and Motor Behavior Department, College of Sport Sciences and Physical Activity, King Saud University, Riyadh 11451, Saudi Arabia; aalsheeri@ksu.edu.sa; 9 Exercise Physiology Department, College of Sport Sciences and Physical Activity, King Saud University, Riyadh 11451, Saudi Arabia

**Keywords:** physical fitness, handgrip strength, standing long jump, children, Saudi girls

## Abstract

**Highlights:**

**What are the main findings?**

- Handgrip strength (HGS) was independently and positively associated with standing long-jump (SLJ) performance in Saudi schoolgirls aged 10–14 years.
- HGS was not significantly associated with 20-m shuttle-run test (20mSRT) performance.

**What are the implications of the main findings?**

- HGS may reflect aspects of lower-limb explosive performance in schoolgirls.
- HGS alone may not adequately reflect cardiorespiratory fitness.

**Abstract:**

**Objective:** This study examined the associations between aerobic and muscular fitness and evaluated whether handgrip strength (HGS) could serve as a simple indicator of physical fitness, as determined by physical fitness tests, among Saudi girls aged 10 to 14 years. **Methods:** Four hundred and fifty-seven girls aged 10 to 14 years from elementary and intermediate schools in Buraidah City, Saudi Arabia, were selected to participate in this cross-sectional study. Anthropometric measurements (height, weight, body composition, and fat percentage) were assessed. Physical fitness tests included HGS, standing long jump, and a 20-m shuttle run. **Results:** Absolute HGS was positively and significantly correlated with all study variables, except the 20mSRT. Absolute HGS was positively correlated with age, height, weight, BMI, fat percentage, and SLJ performance, but not with 20mSRT performance. In mixed-effects regression models that accounted for clustering by school, age, BMI, and HGS were not significantly associated with 20mSRT performance. In contrast, HGS was independently and positively associated with SLJ performance (B = 2.23 cm per kg HGS, 95% CI 1.62 to 2.84, *p* < 0.001), whereas age and BMI were negatively associated with SLJ. Similar findings were observed in analyses stratified by elementary and intermediate school levels, in which HGS remained a significant positive predictor of SLJ but not of 20mSRT performance. **Conclusions:** HGS was independently associated with SLJ but not with 20mSRT performance. These findings indicate that HGS was independently associated with standing long-jump performance among Saudi schoolgirls.

## 1. Introduction

Health-related physical fitness is an important component of children’s growth and development, encompassing cardiorespiratory and muscular fitness, which are important components of physical fitness in children and adolescents. The 20-m shuttle run test (20mSRT) is a valid test of cardiovascular fitness among boys and girls aged between 10 and 16 years old [1]. Alongside aerobic capacity, muscular fitness represents a key component of health-related physical fitness in children and adolescents. It provides important benefits for musculoskeletal health and functional performance when appropriately developed through structured activities [2].

Interactions between components of musculoskeletal fitness and cardiorespiratory fitness have been examined in youth populations. Evidence indicates that these domains are related, typically showing low-to-moderate correlations [3]. For example, musculoskeletal fitness (MSF) positively correlated with aerobic capacity, but at a low-to-moderate correlation strength [4]. Field-based tests of lower limb function, such as the standing long jump (SLJ) and the 1 min sit-to-stand test, are commonly used to assess muscular performance in children. Their relationship has been examined among Saudi children, including the potential influence of body mass index (BMI), physical activity, and age [5]. The 1 min Sit-to-Stand test predicted SLJ performance both before (β = 0.55) and after (β = 0.47) adjustment for confounders, indicating an association between lower-body muscular endurance and explosive performance [5]. SLJ was moderately correlated with peak and mean powers over 10 and 30 s of the Wingate test in children, suggesting its potential use as a field-based test of lower-body explosive performance [6].

MSF may differ between boys and girls, with some variations by age. Recent normative data confirm that handgrip strength (HGS), a measure of muscular strength, is consistently higher in boys than girls across childhood and adolescence, with the differences becoming more pronounced with age [7,8]. At adolescence, by age 13–15 years, the differences were pronounced in all muscular tests [4]. Likewise, Australian [9], Canadian [10], and Saudi [8] reference values showed that boys scored higher than girls in aerobic and muscular fitness, with a greater magnitude in their teenage years.

However, beyond these general sex differences, maturational development is accompanied by changes in body size and composition, which may influence muscular strength and physical fitness [11]. Body mass mediated the positive relationship between biological maturation and HGS in prepubescent girls. Similarly, lean mass was found to mediate the relationship between BMI and HGS in adolescents, highlighting the potential role of lean mass in muscular strength [12]. Moreover, fat mass and fat-free mass have been associated with physical fitness in adolescent girls [13].

In this context, anthropometry and body composition have been proposed to interact strongly with physical fitness in childhood. For example, weight status and physical characteristics were found to predict MSF development, and such potential impacts are important to understand children’s physical development [14]. BMI was associated with the fat-to-lean body mass ratio and was negatively associated with cardiorespiratory endurance, flexibility, and the push-up test in Saudi prepubescent boys and girls [15]. Several independent determinants were examined, and age, sex, BMI, and physical activity were strong determinants of physical fitness in children. Specifically, BMI was the strongest determinant in longitudinal analysis, independent of physical activity [16]. It was found that BMI had negative correlations with weight-bearing exercises, such as 20mSRT and lower-extremity muscular tests. In contrast, it had a positive correlation with HGS and an inconsistent direction for joint mobility and flexibility in South African girls [17].

Previous work suggests that HGS is associated with standing long jump performance and lower limb function [18]. Similar associations between HGS and measures of lower limb performance have also been reported in children and adolescents [18]. In addition, Richardson et al. demonstrated significant associations between HGS, skeletal muscle mass, peak oxygen consumption, and peak work rate in youth, highlighting the broader relationship between HGS and physical fitness [19]. As noted above, the curves of aerobic and muscular development during childhood and adolescence may differ between boys and girls, particularly at the pre-adolescent stage. However, whether the associations between HGS and physical fitness reported among Saudi boys are also observed among Saudi girls remains underexplored. Therefore, the present study aimed to extend this investigation to Saudi girls aged 10–14 years by examining whether HGS was independently associated with standing long-jump and 20-m shuttle-run performance after accounting for age, BMI, and clustering of participants within schools.

## 2. Materials and Methods

### 2.1. Study Design and Participants

This cross-sectional study took place in Buraidah, Al-Qassim region, Saudi Arabia, using a multistage stratified cluster design. This design considered both geographic areas within the city and school type (private or state). Twelve schools (six elementary and six intermediate) were randomly chosen from the three main educational branch offices in Buraidah: East, West, and Central. The Ministry of Education gave permission to conduct the study in these schools. The study was conducted between December 2024 and April 2025.

All female students aged 10 to 14 years who were recruited in girls’ elementary and intermediate schools during the study period were eligible to participate. Students with physical impairments or medical conditions that affected their ability to engage in physical activity, such as eating disorders, cardiovascular disease, or type 1 diabetes mellitus, were excluded.

The study was approved by the Institutional Review Board of King Saud University (IRB No. KSU-HE-24-682). Written informed consent was obtained from the parents or legal guardians of all participants prior to data collection. Assent was obtained from all participating students after they received age-appropriate information about the study. Participants and their parents or guardians were informed that participation was voluntary and that participants could withdraw from the study at any time without penalty. Formal letters were sent to participating schools to obtain institutional cooperation and facilitate study implementation. The school administration selected the classes and invited students, rather than the research team. Students were included in the study if they met the eligibility criteria and the required consent was provided. A total of 457 female students from 12 schools provided parental consent and completed all required fitness assessments. No participants were excluded after consent. There were no missing observations for age, height, weight, BMI, body fat percentage, HGS, 20-m shuttle-run performance, or standing long-jump performance; thus, complete data were available for all 457 participants.

The sample size was initially estimated based on an assumed population proportion of 50% (*p* = 0.50), a 95% confidence level, and a 5% margin of error. An additional 5% was allowed for potential non-responses and incomplete data, resulting in a minimum target sample of approximately 404 students. As a supplementary analysis, G*Power software (V.3.1.9.7) was used to estimate the sample size for a multiple linear regression model, assuming a medium effect size (f^2^ = 0.15), α = 0.05, 95% power, and three predictors (age, BMI, and HGS). This analysis yielded a minimum sample size of 119 participants. This estimate was then adjusted to account for the clustering of students within schools using the design effect, $\mathrm{DE}=1+\left(\bar{m}-1\right)\rho$, where $\bar{m}$ represents the average number of students per school and *ρ* represents the assumed intraclass correlation coefficient (ICC). This method was described by Snijders [20] and applied in a previous study using linear-mixed effects models [21]. With approximately 33 students per school and an assumed ICC 0.05, the design effect was 2.63, increasing the required sample size to approximately 313 students. The final sample size comprised 457 students from 12 schools, exceeding the estimated minimum requirement.

### 2.2. Study Measurements

All assessments were performed on school days between 7:30 and 11:00 a.m. using standardized assessment protocols. Anthropometric measurements, the HGS test, and the standing long jump test were performed by the main researcher. The 20-m shuttle run test was performed by the principal researcher, assisted by a research assistant, when the number of participating students exceeded five to ensure appropriate test administration and participant safety. Assessments were conducted in the following order: body weight and height measurements, HGS, standing long jump, and 20-m shuttle run.

#### 2.2.1. Anthropometry Assessment:

Anthropometric and body composition measurements were conducted during a standardized morning assessment period, in the first two hours of the school day, approximately between 7:30 and 9:00 a.m. Height was recorded to the nearest centimeter with a calibrated rod, with participants positioned barefoot. Body weight and composition were evaluated using bioelectrical impedance analysis with a hand-to-foot scale (Omron BF-511). Before each measurement, the participant’s height, age, and sex were entered into the device. The manufacturer indicates that the device is suitable for children aged 6 years and older, and the Omron BF-511 has been used in previous studies involving children and adolescents, including the NW-CHILD study [22] and school children from Saudi Arabia [23]. Measurements were recorded after entering each student’s height, age, and sex into the apparatus. Participants were instructed not to consume heavy meals within three hours before measurement, to maintain normal hydration, and to avoid performing physical exercise [24]. Female students were also instructed to void their bladders immediately before the assessment, rest quietly for about 10–15 min, and remove any jewelry to minimize potential effects on the bioelectrical impedance measurements. Participants were assessed barefoot and attired in light apparel, adhering to the manufacturer’s specifications. BMI is determined by dividing weight in kilograms by the square of height in meters. Moreover, body fat % serves as an index for body composition in statistical analyses.

#### 2.2.2. HGS

An isometric handgrip dynamometer (Baseline^®^ Smedley spring-typedynamometer, Fabrication Enterprises, Inc., White Plains, NY, USA) was utilized to evaluate muscular strength, which has been previously examined for validity and reliability [25]. Hand dominance was determined by asking students about the hand they use for handwriting. Both trials were performed using the participant’s dominant hand. The dynamometer handle was adjusted to fit each participant’s hand, allowing four fingers to wrap comfortably around it. While standing, participants kept their heads straight, eyes forward, arms hanging alongside the body, wrists in a natural position, feet about hip-width apart, chest up, shoulders back, and torso upright. A familiarization trial was performed using submaximal force before the test. Female students grasped the dynamometer and exerted maximal force in two trials for 4–5 s per trial, with verbal encouragement. A 60-s rest interval was provided between trials, and the highest peak force recorded across the two trials was retained for analysis. Peak force was recorded in kilograms (kg). The procedure followed a standard operating procedure manual used in our laboratories, which was primarily based on the Centers for Disease Control and Prevention’s National Health and Nutrition Examination Survey [26].

#### 2.2.3. Aerobic Fitness

The participants’ aerobic fitness was assessed using the FITNESSGRAM PACER (Progressive Aerobic Cardiovascular Endurance Run), a modified version of the 20-m shuttle-run test (20mSRT) [27]. This test was previously used among Saudi children and adolescents [28,29]. All assessments were conducted on flat, non-slip surfaces in indoor sports halls during physical education classes and were supervised by the main researcher and physical education teachers. Before testing, participants completed a standardized warm-up consisting of light jogging and dynamic movements for approximately 5 min under the supervision of the research team. A maximum of five participants were tested simultaneously. During the test, participants needed to continuously run back and forth as long as possible between two lines separated by 20 m, following the researcher’s instructions and the PACER audio signals. The starting speed was 8.5 km/h and increased by 0.5 km/h each minute. Participants completed the test in small groups of 4–5 students. A trained research assistant monitored participant performance, recorded completed shuttle counts, and assisted with test administration to ensure standardized procedures. The test was terminated when participants failed to reach each line before the audio signal on two occasions or due to volitional fatigue. The main researcher recorded the total number of completed laps as the final score.

#### 2.2.4. Muscular Strength

Explosive muscular strength of the lower body was measured using the Standing long jump test (SLJ), which was conducted on a hard surface. The researchers and physical education teachers supervised the test to ensure participants’ safety and standardize data collection. Participants stood behind a marked starting line with their feet parallel. Participants were required to jump forward as far as possible and land with both feet simultaneously, using an arm swing and knee flexion to increase the velocity of the body’s center of gravity [29,30]. A three-minute interval rest was provided between the two trials to minimize fatigue [5]. The distance from the take-off line to the nearest heel mark at landing was measured, and the longest distance of the two trials was recorded in centimeters [31].

### 2.3. Statistical Methods

The collected data were cleaned, organized, coded, and analyzed using the Statistical Package for the Social Sciences (SPSS Inc., Chicago, IL, USA) version 26. Descriptive statistics are presented as mean ± standard deviation (SD) for normally distributed variables and median with (25th–75th) percentiles for non-normally distributed variables. Between-group differences were evaluated using independent samples *t*-tests for normally distributed variables and the Mann–Whitney U test for non-normally distributed variables. Categorical variables, when applicable, were compared using the chi-square test. Associations between study variables were examined using Pearson correlation coefficients for both the overall sample and stratified school groups (elementary and intermediate). School-level subgroup analyses were exploratory and were performed as an educational classification of the study sample rather than as a measure of biological maturation. There were no missing data for the variables included in the analyses; therefore, no imputation or other missing-data procedures were required, and all 457 participants were included in the statistical analyses. Linear mixed-effect model analyses were performed to identify independent predictors of 20mSRT and SLJ. Linear mixed-effects models were fitted in R (version 4.6.1 www.r-project.org) using the lme4 package (version 2.0-6) and estimated using restricted maximum likelihood (REML). Degrees of freedom and *p*-values for the fixed effects were obtained using the Satterthwaite approximation implemented in lmerTest (version 3.2-1). Given the limited number of schools, the robustness of the findings was additionally examined using the Kenward–Roger small-sample correction implemented in pbkrtest (version 0.5.5). All models were checked using graphical and statistical diagnostics. Normality of residuals was assessed using Q–Q plots and the Shapiro–Wilk test, while linearity and homoscedasticity were assessed using residual plots and the Breusch-Pagan test. Standardized residuals and Cook’s distance were used to examine extreme and influential observations. For the 20mSRT, the original Lap model showed high residual skewness (6.095), excess kurtosis (60.079), and heteroscedasticity (*p* < 0.001). Therefore, Lap was natural-log transformed. There were no zero-lap observations. After transformation, the model diagnostics improved, with lower residual skewness (0.393) and excess kurtosis (2.464) and no evidence of heteroscedasticity based on the prespecified criterion (*p* = 0.012). The Q–Q plot also showed an improved residual distribution, although some deviations remained at the tails. Accordingly, regression coefficients (B) and their 95% confidence intervals are reported on the log scale. Back-transformation was not applied, and nonsignificant associations were not further interpreted in the results. No transformation was required for SLJ. All statistical tests were two-tailed, and the statistical significance was defined as a *p*-value < 0.05.

## 3. Results

Characteristics of participants in body composition and physical fitness tests by age group are shown in Table 1. The mean age of the participants was 11.88 ± 1.31 years; mean height was 144.90 ± 8.74 cm; mean weight was 46.30 ± 12.92 kg; and mean BMI was 21.84 ± 4.93 kg/m^2^. Mean HGS was 16.13 ± 4.59 kg, and the body fat percentage was 27.27% ± 11.10%. The median 20m shuttle run test (20mSRT) performance was 8.0 laps (25th–75th percentiles: 6–12), and the mean standing long jump (SLJ) distance was 131.95 ± 31.36 cm.

Correlation analyses in Table 2 showed that absolute HGS was significantly and positively correlated with age, weight, height, BMI, body fat percentage, and standing long jump (SLJ), with the coefficients ranging from 0.223 to 0.550. There was no significant correlation between HGS and the 20-m shuttle run test (20mSRT; r = −0.048). HGS exhibited negligible correlations with 20mSRT and significant positive associations with SLJ. HGS showed significant positive correlations with age, weight, height, BMI, body fat percentage, and SLJ in both school groups. The strongest correlation was with height among elementary students (r = 0.595) and with weight among intermediate students (r = 0.437). In contrast, HGS was not significantly correlated with 20mSRT in either group.

Table 3 presents the linear mixed-effects regression analysis of the 20mSRT (laps) and SLJ for the overall sample. School was included as a random intercept to account for clustering among participants within schools.

In the 20mSRT model, age, BMI, and HGS were not significantly associated with performance (all *p* > 0.05). The model explained a very small proportion of the variance (marginal R^2^ = 0.003) with an ICC of 0.160.

**Figure 1:**
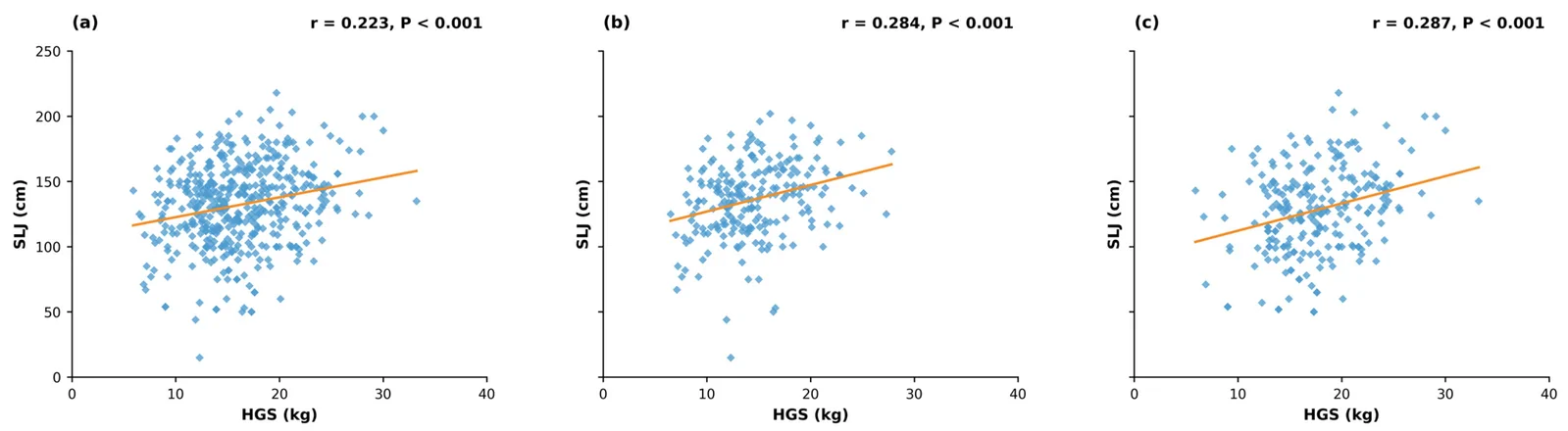
Association between handgrip strength (HGS) and standing long jump (SLJ) in centimeters among female children; (**a**) represents all participants, (**b**) represents Elementary School, and (**c**) represents Intermediate School.

Age and BMI were significant negative predictors for the SLJ model, while HGS was a significant positive predictor. Age (B = −4.050, *p* = 0.015) and BMI (B = −1.069, *p* < 0.001) were negatively associated with SLJ performance, while HGS was positively associated with jump distance (B = 2.230, *p* < 0.001). The corresponding unadjusted trends are shown in Figure 1. The model had an R^2^ of 0.092 (marginal) and an ICC of 0.236.

In the mixed-effects analysis (Table 4), HGS was not significantly associated with 20mSRT in either elementary or intermediate school students. In contrast, HGS was positively associated with SLJ in both elementary and intermediate school students (B = 2.690 and 1.901, respectively; both *p* < 0.001). BMI was negatively associated with SLJ in both elementary (B = −1.153, *p* < 0.003) and intermediate (B = −0.981, *p* = 0.011) school students. Age was negatively associated with SLJ among elementary school students (B = −5.481, *p* < 0.043). The ICC values ranged from 0.005 to 0.293, indicating some clustering of performance within schools.

## 4. Discussion

This study investigated the associations between HGS and muscular and cardiorespiratory fitness among Saudi schoolgirls, while accounting for age, BMI, and school-level clustering. The principal finding was that HGS was independently associated with standing long-jump performance but not with 20-m shuttle-run performance. In the overall mixed-effects model, greater HGS was associated with longer jump distance, whereas higher BMI and older age were associated with poorer standing long-jump performance. In contrast, none of the examined predictors significantly explained variation in 20-m shuttle-run performance. These findings indicate that HGS was independently associated with standing long-jump performance, whereas a significant association with 20-m shuttle-run performance was not detected in girls aged 10–14 years. Differences observed between school-level subgroup analyses should not be interpreted as evidence of effect modification.

SLJ is a field test of explosive lower limb performance and is influenced by whole-body coordination and momentum transfer from the upper body and trunk [32]. The positive HGS coefficient across all standing long-jump models suggests that greater overall strength was associated with more effective force generation and transmission during lower-body movements and may reflect shared neuromuscular characteristics underlying upper- and lower-body performance [18,33]. This agrees with reports that SLJ correlates with indices of muscular power in children and that global strength capacities contribute to jumping tasks that recruit multiple segments, including upper limbs and trunk, for impulse generation and landing control [34,35]. These findings suggest that HGS is associated with SLJ performance in school-aged children. However, the modest strength of the association indicates that the two measures should not be considered interchangeable.

The 20-m shuttle-run test is widely used as a field-based assessment of cardiorespiratory fitness and demonstrates acceptable validity for estimating aerobic capacity in youth [1,36,37]. However, shuttle-run performance is influenced by a variety of factors beyond muscular strength, including pacing ability, motivation, movement efficiency, familiarity with testing procedures, and biological maturation. In the present study, neither HGS, age, nor BMI was significantly associated with 20mSRT performance after accounting for school-level clustering, and the model explained only a very small proportion of the variance. These findings suggest that HGS does not capture the factors contributing to 20-m shuttle-run performance in Saudi schoolgirls and support previous evidence indicating that muscular fitness and cardiorespiratory fitness represent related but distinct domains of physical fitness in youth [3].

BMI was negatively associated with standing long-jump performance in the overall model and among elementary school students, whereas no significant association was observed with 20mSRT performance. The inverse relationship with standing long-jump performance is consistent with reports that greater body mass may compromise performance in explosive weight-bearing activities by increasing the mechanical and energetic costs of movement [38,39]. The absence of a significant association between BMI and 20mSRT performance suggests that other physiological and behavioral factors may have played a larger role in shuttle-run performance within this cohort.

Age was negatively associated with standing long-jump performance in the overall model and remained significantly associated with lower performance among elementary school students, whereas it was not significantly associated with performance among intermediate school students. School-level subgroup analyses were exploratory and were not intended to reflect biological maturation status. Interpret this finding cautiously because chronological age may not fully reflect biological maturation during adolescence. Variability in pubertal timing can influence body composition, neuromuscular development, and physical performance independently of chronological age [40]. The validity and interpretation of standing long-jump performance in children are therefore influenced by both maturational and behavioral factors, including motivation and test execution [41]. These findings are consistent with previous studies reporting associations between HGS and measures of lower-limb power in children and adolescents [42,43].

The major strengths of this study include the relatively large and geographically diverse school sample, standardized testing of widely used field-based measures, and the application of mixed-effects regression models that accounted for clustering of participants within schools. Simultaneously considering HGS, age, and BMI allowed us to evaluate their independent associations with physical fitness outcomes. This study has several limitations. Its cross-sectional design precludes causal inference, and the single-region sample may limit generalizability. Body composition was assessed using bioelectrical impedance analysis rather than gold-standard methods. In addition, factors such as physical activity, nutrition, maturation status, and socioeconomic characteristics were not evaluated and may have influenced the findings. School-level subgroup analyses were exploratory and should be interpreted cautiously. Despite these limitations, the relatively large sample provides novel insights into the relationship between body composition and lower-limb muscular fitness in school-aged children.

Biological maturation was not assessed, which is important because pubertal differences may influence muscular performance independently of chronological age [44]. We also did not evaluate physical activity and other potential confounders, including sports participation and motor competence. Consequently, residual confounding cannot be excluded. Finally, the inclusion of girls only may limit the generalizability of the findings to boys and mixed-sex populations.

In conclusion, HGS was independently associated with standing long-jump performance but not with 20-m shuttle-run performance among Saudi schoolgirls aged 10–14 years. Higher BMI and older age were associated with poorer standing long-jump performance in the overall sample, whereas none of the examined predictors were significantly associated with 20-m shuttle-run performance. These findings indicate that HGS is associated with standing long-jump performance but not with 20-m shuttle-run performance in Saudi schoolgirls. Future longitudinal studies incorporating objective measures of biological maturation, habitual physical activity, and aerobic capacity are warranted to clarify the mechanisms underlying these associations.

## Figures and Tables

**Table 1:** Characteristics of the participants in body composition and physical fitness tests.

Parameters	All (n = 457)	Elementary (n = 219)	Intermediate (n = 238)	*p*-Value	Effect Size
Age (years)	11.88 ± 1.31	10.75 ± 0.73	12.92 ± 0.74	<0.001	2.94
Height (cm)	144.90 ± 8.74	139.89 ± 7.81	149.51 ± 6.79	<0.001	1.32
Weight (kg)	46.30 ± 12.92	41.63 ± 11.73	50.60 ± 12.47	<0.001	0.74
BMI (kg/m^2^)	21.84 ± 4.93	21.07 ± 4.87	22.54 ± 4.89	0.001	0.30
HGS (kg)	16.13 ± 4.59	14.49 ± 4.11	17.65 ± 4.49	<0.001	0.73
% Fat	27.27 ± 11.10	23.68 ± 11.79	30.58 ± 9.29	<0.001	0.65
20mSRT (laps)	8.00 (6–12)	8.0 (6–11)	9.0 (6.25–12)	0.153	0.07
SLJ (cm)	131.95 ± 31.36	136.01 ± 29.35	128.21 ± 32.73	0.008	0.25

Note: Data presented as mean ± SD and median (25th–75th) percentiles for Gaussian and non-Gaussian variables. Effect sizes are presented as Cohen’s d for independent-samples *t*-tests and r for the Mann–Whitney U test. *p*-value < 0.05 and <0.01 levels are considered significant. BMI: body mass index; HGS: handgrip strength; % Fat: fat percentage; 20mSRT: 20-m shuttle run test; SLJ: standing long jump.

**Table 2:** Correlations of HGS with study parameters.

Parameters	All (n = 457)	Elementary (n = 219)	Intermediate (n = 238)
	HGS (kg)	HGS (kg)	HGS (kg)
Age (years)	0.415 **	0.342 **	0.165 *
Weight (kg)	0.510 **	0.453 **	0.437 **
Height (cm)	0.550 **	0.595 **	0.334 **
BMI (kg/m^2^)	0.337 **	0.259 **	0.351 **
% Fat	0.433 **	0.324 **	0.418 **
20mSRT (laps)	−0.048	−0.047	−0.042
SLJ (cm)	0.223 **	0.284 **	0.287 **

Note: Data presented as Pearson’s correlation coefficient (r). * *p* < 0.05; ** *p* < 0.01 level. HGS: handgrip strength; BMI: body mass index; % Fat: fat percentage; 20mSRT: 20-m shuttle run test; SLJ: standing long jump.

**Table 3:** Regression mixed-effect analysis of 20mSRT (log-transformed laps) and SLJ for all children.

Outcome	Predictor	B	SE	t	df	*p*-Value	95% CI
20mSRT (log-laps)	Intercept	2.366	0.385	6.144	146.920	<0.001	[1.605, 3.127]
	Age	−0.006	0.033	−0.185	177.682	0.853	[−0.071, 0.059]
	BMI	−0.007	0.005	−1.298	449.334	0.195	[−0.017, 0.003]
	HGS	0.003	0.006	0.447	447.440	0.655	[−0.009, 0.015]
	ICC	0.160					
	R^2^	0.003					
SLJ (cm)	Intercept	170.614	19.777	8.627	208.307	<0.001	[131.625, 209.603]
	Age	−4.050	1.662	−2.437	274.268	0.015	[−7.322, −0.778]
	BMI	−1.069	0.269	−3.981	447.468	<0.001	[−1.598, −0.540]
	HGS	2.230	0.311	7.171	445.988	<0.001	[1.619, 2.841]
	ICC	0.236					
	R^2^	0.092					

Note. Linear mixed-effects models included school as a random intercept to account for clustering of participants within schools. B: unstandardized regression coefficient; SE: standard error; CI: confidence interval; 20mSRT: 20-m shuttle run test; BMI: body mass index; HGS: handgrip strength; SLJ: standing long jump; ICC: intraclass correlation coefficient. Lap was modeled on the natural-log scale; R^2^: Marginal R^2^.

**Table 4:** Regression mixed-effect analysis of 20mSRT (log-transformed laps) and SLJ by school group.

School	Outcome	Predictor	B	SE	t	df	*p*-Value	95% CI
Elementary	20mSRT (log-laps)	Intercept	2.128	0.602	3.533	200.420	<0.001	[0.940, 3.315]
		Age	0.033	0.056	0.594	213.918	0.553	[−0.077, 0.143]
		BMI	−0.010	0.008	−1.301	212.324	0.195	[−0.026, 0.005]
		HGS	−0.005	0.010	−0.492	212.107	0.623	[−0.025, 0.015]
		ICC	0.190					
		R^2^	0.009					
	SLJ (cm)	Intercept	181.796	28.695	6.335	206.501	<0.001	[125.222, 238.369]
		Age	−5.481	2.694	−2.035	214.952	0.043	[−10.790, −0.172]
		BMI	−1.153	0.380	−3.033	213.506	0.003	[−1.902, −0.404]
		HGS	2.690	0.483	5.569	213.358	<0.001	[1.738, 3.643]
		ICC	0.208					
		R^2^	0.123					
Intermediate	20mSRT (log-laps)	Intercept	2.599	0.618	4.203	232.622	<0.001	[1.381, 3.818]
		Age	0.033	0.048	−0.691	232.384	0.490	[−0.128, 0.062]
		BMI	−0.005	0.008	−0.689	233.999	0.492	[−0.020, 0.010]
		HGS	0.007	0.008	0.856	232.371	0.393	[−0.009, 0.023]
		ICC	0.005					
		R^2^	0.002					
	SLJ (cm)	Intercept	160.825	31.776	5.061	217.563	<0.001	[98.198, 223.453]
		Age	−3.040	2.428	−1.252	232.071	0.212	[−7.824, 1.745]
		BMI	−0.981	0.382	−2.566	231.026	0.011	[−1.734, −0.228]
		HGS	1.901	0.412	4.612	229.909	<0.001	[1.089, 2.713]
		ICC	0.293					
		R^2^	0.063					

Note. Linear mixed-effects models included school as a random intercept to account for clustering of participants within schools. B: unstandardized regression coefficient; SE: standard error; CI: confidence interval; 20mSRT: 20-m shuttle run test; BMI: body mass index; HGS: handgrip strength; SLJ: standing long jump; ICC: intraclass correlation coefficient. Lap was modeled on the natural-log scale; R^2^: Marginal R^2^.

## Data Availability

The data presented in this study are available from the corresponding author upon reasonable request due to ethical considerations and the protection of participants’ privacy.
